# Vorbeireden. Could it be Ganser Syndrome?

**DOI:** 10.1192/j.eurpsy.2023.937

**Published:** 2023-07-19

**Authors:** J. Petta, A. L. Falcão, G. Soares, A. Lourenço

**Affiliations:** Centro Hospitalar Psiquiátrico de Lisboa, Lisboa, Portugal

## Abstract

**Introduction:**

Ganser syndrome is described as a dissociative disorder not otherwise specified in the DSM-IV, and is not currently listed in the DSM-V.

It is a rare condition, with transient Vorbeireden as the central symptom. This means the patient responds to questions with an incorrect answer, but by the nature of the answer reveals an understanding of the question posed.

This disorder was first described by the German psychiatrist Sigbert Ganser in 1898.

**Objectives:**

Analyze case reports published in the available literature and intelligibly characterize their clinical presentation and dissect the etiopathogenesis of the disease.

**Methods:**

Data was obtained through an internet-based literature search, using the databases PubMed, Cochrane Library and NCBI. The World Health Organization was also utilized. Seven articles from the last four years were included.

**Results:**

The core clinical features of this syndrome are approximate answers, clouding of consciousness, somatic conversion symptoms and hallucinations. However, they are all not needed for diagnosis.

The basic underlying etiology of Ganser syndrome is still unknown. Debates over the factitious versus psychiatric versus organic origin of the symptomatology are common in the literature.

No reliable epidemiological data can be established.

**Conclusions:**

The condition is a rare, probably dissociative, with transient *Vorbeireden* as the central symptom.

Although the research interest in dissociative disorders, the etiopathogenetic models remain hypothetical. Detailed imaging, neuropsychological and neurological data are required.

**Disclosure of Interest:**

None Declared

